# A Scoping Review of the Serious Game-Based Rehabilitation of People with Cerebral Palsy

**DOI:** 10.3390/ijerph20217006

**Published:** 2023-11-01

**Authors:** Si Nae Ahn

**Affiliations:** Department of Occupational Therapy, Cheongju University, Cheongju 28503, Republic of Korea; otlovesn@gmail.com or otlovesn@cju.ac.kr

**Keywords:** cerebral palsy, game, intervention, rehabilitation, review, serious context

## Abstract

In a serious context, individuals with Cerebral Palsy (CP) have limited opportunities to engage in social interaction experiences. Through a review, this study provides an explanation and improved evidence of the methods for rehabilitation in games used in serious contexts for people with CP. Articles published from 2010 to 2022 focusing on serious game-based rehabilitation for people with CP are extracted from MEDILINE, Academic Search Ultimate, CINAHL, and the Web of Science. The articles were assessed based on the McMaster critical review form. This study analyzes the frequencies of goal and assessment tools according to the components using the International Classification of Functioning, Disability and Health (ICF). The evidence of all the studies is presented according to the principles of Population, Intervention, Comparison, Outcome (PICO) to organize the evidence. A total of 19 articles were selected. Five articles involved Randomized Controlled Trials (RCTs), six articles involved non-randomized one-group designs, three articles involved single experimental study designs, and five articles were case report designs. In the selected articles, the average score on the McMaster critical review form was 11.8 points. In the game-based rehabilitation for CP, more articles reported goals and assessment tools focusing on body function than goals and assessment tools focusing on activity and participation, according to the ICF. These findings provide a record of past work and identify the evidence to support the application of game-based rehabilitation for people with CP.

## 1. Introduction

Cerebral Palsy (CP) is characterized by permanent impairments to muscle tone, posture, and movement, and may lead to mental disability and functional alterations, which are often accompanied by disturbances in sensation, perception, cognition, communication, and behavior [1,2,3]. The abnormalities in brain development associated with CP have also been found to cause several functional and physical limitations, leading to difficulties in performing the activities of daily living, education, play, and social participation throughout life [4,5]. CP is one of the leading causes of childhood disability, with 50 million people worldwide living with it [6]. An important component of CP rehabilitation programs is the practice of useful and repetitive movements, as the degree of improvement depends on the level of appropriate activity performed [7]. It has been reported that the repeated and intensive practice of motor tasks promotes functional recovery and stimulates the self-directed realization of practiced skills [8,9]. Addressing these motor and cognitive disabilities usually requires multi-faceted treatment strategies that involve intensive and long-term training [10]. As the performance improves, simple tasks using functional patterns and routines are combined with functional movements that are practiced repetitively. However, such approaches may be resource-intensive and exhausting during long-term rehabilitation processes [11]. Recently, the intervention of games used in severe situations has been proposed as an interesting tool for neurological and cognitive rehabilitation in children with CP. According to previous studies, the adherence to rehabilitation programs determines the motor and functional outcomes for children with CP. Therefore, there is a need for innovative, cost-effective approaches that motivate children with CP to complete long-term rehabilitation programs to improve their neurodevelopmental outcomes [12]. An emerging, promising approach is the integration of enjoyable computer games with a variety of interactive physical and cognitive stimuli that help children actively engage in activities. Several studies have reported the benefits of serious games used as interventions in rehabilitation training [13,14,15]. The ultimate goal of serious games is to allow participants to achieve specific goals in a fun and engaging way [13]. Serious games are games designed to convey learning and encourage certain skills. However, the literature reports the use of commercial Off-The-Shelf (COTS) games to improve general or transverse skills or for rehabilitation purposes [16]. In the healthcare field, motivational decline often occurs in people with CP due to repetitive, intensive activities in rehabilitation programs [17]. Patient motivation is especially important in long-term rehabilitation practices to maintain function and skills. Interventions with games used in serious contexts mainly focus on treatment, recovery, and rehabilitation. They are considered promising solutions for their capability to motivate patients during therapy sessions [18]. Compared with normally developed children, children with CP have limited play and social interaction experiences [19]. Therefore, games provide opportunities for children with CP to engage in serious contexts that are normally inaccessible to them. While the findings of the previous studies show the potential of serious games-based rehabilitation in children with CP [20], there is a lack of clinical evidence. Furthermore, it is also difficult to compare the studies due to the lack of standard rehabilitation strategies and clinical assessments and scales.

A previous review shows moderate evidence that virtual reality rehabilitation is a promising intervention to improve balance and motor skills in children and adolescents with cerebral palsy. In future studies, as this technology develops, long-term follow-up and additional studies are needed to determine its precise position in cerebral palsy management [21]. Consequently, there is insufficient evidence on the positive effects of interventions in games used in serious contexts as a treatment approach for people with CP; therefore, long-term follow-up studies and further research are required to determine this. Therefore, the purpose of this study is to review the literature regarding the application of serious game-based rehabilitation for people with CP. This study aims to present an in-depth understanding of the role of games used in serious contexts for rehabilitation or therapeutic purposes of persons with CP based on the characteristics of the intervention, the types of evaluation, and the outcomes. Through a review, this study provides an explanation for the record of previous studies and improved evidence of methods for serious game-based rehabilitation for people with CP.

## 2. Materials and Methods

### 2.1. Study Design

This review was conducted according to the Preferred Reporting Items for Systematic Reviews and Meta-Analyses Extension for Scoping Reviews (PRISMA-ScR) [22].

### 2.2. Search Strategy

This study reviewed the platforms used for rehabilitation. Articles on serious games using computer programs published from 1 January 2010 to 30 April 2022 were searched in 3 databases: MEDILINE, Academic Search Ultimate, CINAHL, and the Web of Science. A combination of the following keyword search constructs was used: “cerebral palsy” AND “serious game”, “cerebral palsy” AND “video-based game”, “cerebral palsy” AND “computer-based game”, “cerebral palsy” AND “game-based”, “CP” AND “serious game”, “CP” AND “video-based game”, “CP” AND “computer-based game” and “CP” AND “game-based”.

### 2.3. Study Selection

The inclusion criteria were as follows: studies with the original text written in English; studies involving participants with CP; studies including an experimental study design with an intervention; and studies including function or activity and participation evaluations according to the International Classification of Functioning, Disability and Health, known more commonly as ICF, which is a classification of health and health-related domains [23]. The exclusion criteria were as follows: studies based on animals; studies that did not present an intervention (e.g., review articles, longitudinal studies, qualitative studies, and cross-sectional studies); studies that did not contain function or activity and participation evaluations according to the ICF; studies using drugs, injections, and acupuncture as the intervention; and articles for which the full text was not provided. Only publications in English were included.

### 2.4. Searching Process

Original articles that met the inclusion and exclusion criteria were extracted. Using the search terms, a total of 7511 articles were identified. First, through the title and abstract review, articles for which the full text was not provided and duplicate papers were excluded according to the exclusion criteria. Second, articles meeting the inclusion and exclusion criteria were selected and evaluated through their abstracts. Subsequently, these abstracts and full texts were further scrutinized using the inclusion and exclusion criteria. Studies that could not be included solely using the inclusion criteria or the content of the abstract were selected through a full-text review. The final list for the review consisted of 19 studies.

### 2.5. Data Extraction and Quality Assessments

The collected data included the study, study populations, and intervention characteristics. The characteristics that were extracted were as follows: first author, year of publication, country, follow-up, dropout, and adherence rates; the study population, the type of CP of the participants, and the age of the participants; and the intervention place, intervention type, and structure. The evaluation characteristics were also extracted. An evidence-based classification based on the five stages of evidence presented by Arbesman, Scheer, and Lieberman [24] was used to classify the articles. Level-I studies, including randomized controlled trials (RCTs); level-II studies, in which the assignments to treatment or control groups were not randomized; levelIII studies without a control group; level-IV studies with a single-case experimental design; and level-V studies with case reports were employed [25].

To further examine all the evidence related to serious game interventions, the studies were appraised for their quality, utilizing the McMaster Critical Review Form for quantitative studies. The McMaster Critical Review Form is easy to use and comprehensive. The form was developed by the McMaster University Occupational Therapy Evidence-Based Practice Research Group [26]. In the McMaster Critical Review Form, each study was assessed as to whether it fulfilled the requirements listed under each criterion. If the requirement was satisfied, it was rated “yes” and received a score of one. If the requirement was not or inadequately satisfied, it was rated “no” or “n/a” and received no points. The maximum total score for the quantitative studies was 15 points [27].

## 3. Results

### 3.1. Search Results

**Figure 1:**
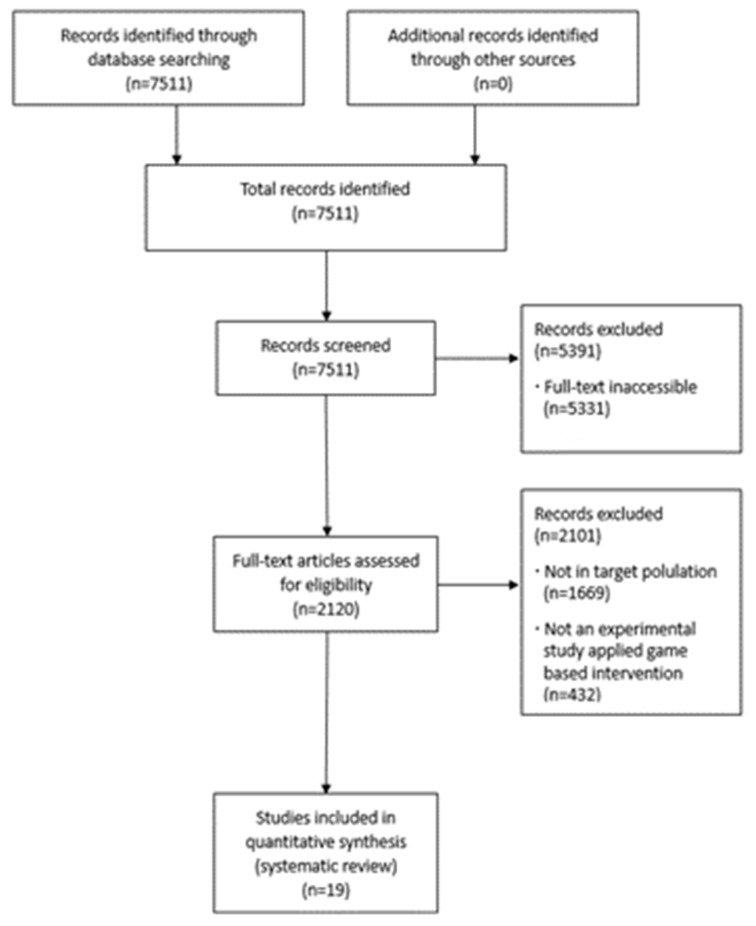
Flow diagram.

Through a search of the three databases, 7511 articles were initially identified. In the first screening, 7511 records were screened based on their titles and abstracts. Of these, 71.0% (n = 5391) were excluded based on the full-text accessibility and duplicability, and the remaining 29.0% (n = 2120) proceeded to full-text screening. According to the eligibility criteria, 2101 articles were excluded for the following reasons: the primary diagnosis was something other than CP (n = 1669); the experimental study excluded game-based interventions (n = 432). Therefore, 19 articles met the eligibility criteria and were included in the data extraction. The study selection process is summarized in the flow diagram in Figure 1.

### 3.2. Methodological Quality of Studies

The quality of the evidence in the selected articles was assessed using the evidence-based level of classification, the McMaster Critical Review Form. This review analyzed the quality assessment by applying the evidence-based level classification based on the five-stage evidence presented by Arnesman, Scheer, and Liberman [24]. Five articles (26.3%) involved RCTs, six articles (31.6%) involved non-randomized one-group designs, three articles (15.8%) involved a single experimental study design, and five articles (26.3%) had a case report design (Table 1).

Among the RCTs, two [15,28] included a randomized crossover trial study design (Appendix A). Table 2 provides a detailed summary of the results of the assessment of the quality of evidence using the McMaster Critical Review Form. One article completely fulfilled the requirements of each criterion, was assessed as being of a high quality, and achieved 100% with a total score of 15 points, according to the quantified McMaster Guidelines criteria [29]. The remaining articles were assessed as being in the good-quality range; three articles analyzed achieved over 80% with total scores of 13 points [15,30,31], 12 articles achieved over 70% with total scores of between 11 [13,32,33,34,35] and 12 points [14,28,36,37,38,39,40], and one article achieved a total score of 10 points [41]. According to the quantified McMaster Guidelines criteria, eight of the selected articles did not meet the full total score in terms of the sample, intervention, and results.

### 3.3. Population

The nineteen articles comprised a total sample of 202 participants, with the average number of participants being 10.63 ± 8.19 (range 1 to 28) per study. When the age classification of the participants was analyzed, the frequency of articles for preschoolers (2 to 5 years old) was two (10.5%), the frequency of articles for school-age child (6 to 12 years old) was eight (42.1%), the frequency of articles for preschool (2 to 5 years old) to school-age children (6 to 12 years old) was two (10.5%), the frequency for school-age children (6 to 12 years old) to adolescents (13 to 19 years old) was four (21.1%), the frequency for preschoolers (2 to 5 years old) to adolescents (13 to 19 years old) was one (5.3%), and the frequency for adults (over 20 years old) was two (10.5%). When the type of CP was reported, the frequency of articles for spastic hemiplegia was five (26.3%), the frequency for spastic diplegia was one (5.3%), the frequency for spastic hemiplegia and diplegia was two (10.5%), and the frequency for unspecified types was eleven (46.7%) (Appendix A).

### 3.4. Interventions

This study reviewed 19 articles to provide evidence to support interventions in games used in serious contexts for individuals with CP. In 18 articles, the average period of the intervention was 12.38 ± 12.57 (range: 2 to 56) weeks. In one study, the intervention period was 10 sessions; however, the number of weeks was not specified. As a result of the frequency analysis of the intervention location, the frequency of articles at home was three (15.58%), the frequency at school was two (10.5%), and the frequency at rehabilitation centers was fourteen (73.7%) (Appendix A). The intervention goals of the 19 selected articles were classified according to the concept of ICF (Table 3).

Based on the ICF component, the intervention goals for body function were higher than those for activities and participation. Regarding the level of evidence of the study, study designs from levels I to V of evidence were included in the category of body function, and study designs from levels Ⅳ to V of evidence were included in the category of activities and participation. The intervention goals for body function included cervical and trunk control, balance with postural control, movement coupling, sitting ability, bimanual coordination, upper limb function, and visual perceptual skills. Intervention goals for activities and participation included activities, participation, and quality of life.

Regarding the level of evidence of interventions in games used in serious contexts, four articles at level I applied serious game interventions targeting body functions according to the ICF concept [15,29,31,40]. The remaining article of level I focused on body functions along with activities and participation [28]. Sajan et al. reported the effectiveness of interactive video gaming with the Nintendo Wii compared to conventional occupational therapy in setting goals, such as posture control and balance, upper limb function, visual–perceptual skills, and functional mobility in children with CP [29]. Szturm et al. reported a procedure and an acceptable novel game-based dual-task balance exercise program with high adherence and positive outcomes in children with CP [40]. Velasco et al. [31] conducted a study on the effects of cervical and trunk control based on serious videogames and physical exercise, comparing it with non-intervention phases in setting goals, such as cervical and trunk control. Wade and Porter [15] reported on the effectiveness of a seat “cushion” that contained a platform and was based on a modified game controller in setting goals, such as sitting ability. The control group received traditional physical and occupational therapy. Zoccolillo et al. performed video-game-based therapy using an Xbox with a Kinect device, which was effective in improving the motor functions of the upper limb extremities in children with CP [28].

Five level-III articles applied serious game interventions targeting body functions according to the ICF concept [4,13,36,37,42]. The remaining level-III articles focused on body functions, activities, and participation [38]. Amengual Alcover et al. reported on PROGame, a novel process framework for the development of serious games for motor rehabilitation in adults with CP [13]. PROGame introduces a structured process in terms of modeling, construction, and validation, and is based on a web application development model. Camara Machado et al. [4] studied an Xbox360 Kinect game used in a rehabilitation program at a hospital, and Jaume-i-Capó et al. reported that serious games for balance rehabilitation therapy demonstrated a significant increase in balance and gait function scores, an indicator of more independence in the participating adults with CP [36]. Keller and Van Hedel reported the effectiveness of weight-supported training in a playful and virtual environment using Armeo^®^ Spring, the exergame Moorhuhn, for children and adolescents with CP [37]. Sandlund et al. reported the effectiveness of a home-based intervention using the EyeToy for PlayStation 2 [42], while Luna-Oliva et al. examined the Kinect Xbox 360 and showed improvements in balance and theactivities of daily living in children with CP in a school setting [38].

Three articles from level IV applied interventions in games used in serious contexts targeting body functions along with activities and participation according to the ICF concept [30,32,39]. Do et al. reported the efficacy of bilateral arm training using the Nintendo Wii game in children with CP [32]. MacIntosh et al. conducted a biofeedback-enhanced therapeutic exercise video game intervention at home for young people with CP [30]. Peper et al. reported the effectiveness of Lissajous-based training on the bimanual performance of children with CP [39].

Two level V articles applied interventions in games used in serious contexts targeting body functions along with activities and participation according to the ICF concept [33,34,41]. The remaining two articles of level V focused on body functions [35,41]. Burdea et al. reported that playing two customized virtual reality games was age-appropriate and well received by the participants, and that game-based ankle robot training was beneficial for gait in children with CP [41]. Moldovan et al. reported on the effectiveness of the MIRA exergames with Xbox^®^ sensors in children with CP [33], and Reifenberg et al. confirmed the feasibility of game-based neurorehabilitation using telemedicine technology [34]. Barton et al. reported on the effectiveness of the Goblin Post Office game on the CAREN virtual rehabilitation system in children with CP [14], and Sanjay et al. conducted a computer game-assisted repetitive task practice based on an upper extremity therapy program in children with CP [35].

### 3.5. Outcome Measurements

Table 3 provides the classification of assessment tools according to the ICF concept. The Gross Motor Function Measure, Quality of Upper Extremity Skills Test, Assisting Hand Assessment, Box and Block Test, Bruininks-Oseretsky Test of Motor Proficiency-2, Balance Tinetti Test, and Walk Test were used to assess body function. Assessment tools for activities and participation included the Pediatric Motor Activity Log, ABILHAND-Kids, Assessment of Motor and Process Skills, Chailey Levels of Ability, Goal Attainment Scale, Pediatric Evaluation and Disability Inventory, and Pediatric Quality of Life Inventory.

## 4. Discussion

This study aimed to provide a record of previous work to support the application of serious game-based rehabilitation for people with CP and, through the review, to improve the understanding of the methods of intervention in the games used. All 19 articles included in this review were studies on individuals with CP. Most articles that applied interventions in games used in serious contexts to persons with CP did not specify the type of CP in the inclusion criteria in the study; however, some specified spastic hemiplegia with CP. According to age classification, most articles recruited participants with children with CP. There were only two articles where adults were included. The types of CP depended on the nature of the injury and the area of involvement [34]. Consequently, serious game interventions need to be applied to many types of CP cases, and their effectiveness in improving independence in activities of daily living, social participation, and overall quality of life still needs to be verified. Moreover, these impairments can lead to lifelong limitations in performing typical occupations, including activities of daily living, school, play, and social participation [5].

The application of interventions in games used in serious contexts were commercially available game programs (e.g., Nintendo Wii, Xbox 360 Kinect, PlayStation, and Goblin Post Office game), frameworks of games that were newly developed (PROGame), those that were combined with existing treatments (e.g., Armeo^®^ Spring with the exergame Moorhuhn, MIRA exergames with Kinect Xbox^®^ sensor), and those that were combined with telephone rehabilitation (game-based neurorehabilitation using telehealth technologies). Regarding the intervention methods, the range of the intervention periods was 2 to 56 weeks, and the place of intervention was at school, home, and rehabilitation centers.

Traditional rehabilitation of CP uses two basic principles that emphasize the normalization of the quality of movement and functional activities, which focus on the development of the skills necessary for the activities of daily living [43,44]. A preliminary review reported that virtual reality rehabilitation showed moderate-effect-size evidence that it was a promising intervention to improve balance and motor skills in children and adolescents with cerebral palsy [21]. In this study, because it was a review that presented the latest evidence on serious games for children with cerebral palsy, the effect size could not be presented. In future studies, as this technology develops, it is necessary to compare the effects of serious games through meta-analyses for CP rehabilitation, and long-term follow-up and additional studies are needed.

Game-based interventions are more appealing than traditional treatment methods and may induce increased motivation and social interaction. Interventions in games used in serious contexts have been demonstrated to be interesting tools for the neurological and cognitive rehabilitation of children with CP [4,45,46]. The essential goal of serious games is to generalize them to improve real-life outcomes. This differs from games designed for entertainment, which are not used in this way. This study aimed to provide evidence to support the application of serious game-based rehabilitation for people with CP and, through the review, to improve the understanding of methods of intervention in the games used.

The essential goal of serious games is to generalize them to improve real-life out-comes. This differs from games designed for entertainment, which are not expected to provide generalized skills or knowledge, such as games that are not designed for skill acquisition. Interventions in games used in serious contexts support the generalization of learning, which focuses on the integration of educational objectives with known, specific, evidence-based game mechanics [47]. In this review, although large-scale studies are still needed to investigate the effectiveness of serious game interventions, this approach appears to have positive effects on persons with CP. This review recommends that the application of game-based rehabilitation in serious contexts should include desirable features for rehabilitation, meaningful and motivational elements, and adaptability feedback to increase the capabilities in persons with CP.

In addition, game-based rehabilitation in serious contexts should include strategy protocols for implementing serious games into rehabilitation programs, and considerations for the development of personalized and adaptive game interventions. In keeping with this trend, interventions in games used in serious contexts may offer the possibility of providing intensive training for long-term rehabilitation in persons with CP. Future studies need more evidence to demonstrate the positive effects of rehabilitation interventions in games used in serious contexts.

As summarized in the McMaster Critical Review Form, one out of 19 studies were in the high-quality target range. All analyzed studies scored highly in reporting the appropriate purpose, literature, design, outcome, and conclusions. However, most studies did not meet the criteria in terms of the samples, interventions, or results. While most studies described their samples and inclusion criteria, a few studies did not describe their sample sizes or the handling of dropout participants. A few studies did not report their results in terms of statistical significance.

In this review, because the study design was not limited to RCTs, a group study design, a single-subject design, and a case study design were included. Most of these study designs did not contain randomization or control groups and did not describe the sample sizes and statistical significance of the results. Because this study included a variety of study designs, we were able to obtain a comprehensive overview of the available evidence. However, the non-randomized study design and large proportion of case reports may potentially introduce bias to the synthesis of the results.

All articles included assessments and interventions for an individual’s physical function, activity, and participation according to the ICF. Regarding the level of evidence for serious gaming interventions, none of the RCTs selected for this study targeted activity and participation along with physical function. However, the effects of serious gaming interventions go beyond simple physical functions, and there is a need to test their effects on physical functions across a wider range of activity and participation domains. Future studies should therefore strive for better methodological quality and aim to provide a more comprehensive understanding of the benefits and limitations of functional games in CP rehabilitation.

This review had several limitations. In this study, the review, including various research designs, was conducted to provide comprehensive information on the research topic. Because the literature was comprehensively selected, only five RCT studies were included in the studies we selected, and the remaining studies had a large proportion of non-randomized designs and case reports. This may have led to biases in the synthesis of the results. Therefore, this should be considered when carefully interpreting the results of this study. In the future, there is a need to supplement these aspects and clearly demonstrate treatment effectiveness through a systematic review and meta-analysis of the RCT studies. Given the broad scope of the research question and the amount of literature on CP, it was not possible to provide detailed descriptions of each study for a direct comparison; however, the general characteristics of the selected articles were provided in the table for a reference and comparison. In addition, studies on the interventions based on activity and participation rather than physical function for game-based rehabilitation interventions are necessary to establish evidence for serious game-based rehabilitation for people with CP. Several studies had small sample sizes, limited geographic areas, or used convenience samples, which may have reduced the generalizability of the results. Other limitations included the exclusion of reviews and unpublished articles. In addition, this review was assessed by a single author and the search and data extraction was performed by a single evaluator. To compensate for these limitations, further research should be conducted by searching more extensively for systematic reviews on individuals with CP performed by two independent evaluators.

## 5. Conclusions

The aim of this study was to provide a record of the previous work and identify the evidence to support the application of game-based rehabilitation for people with CP, and to increase our understanding of serious game-based rehabilitation. All 19 articles included in this review were studies of patients with CP. Five articles were related to Randomized Controlled Trials (RCTs), six articles were related to non-randomized single-group designs, three articles were related to single-experimental study designs, and five articles were related to case report designs. The average score on the McMaster Critical Review Form for the selected papers was 11.8. According to the ICF, in game-based rehabilitation for CP, more articles reported goals and assessment tools focused on physical function than goals and assessment tools focused on activity and participation. These findings provide evidence that serious game-based rehabilitation is a potential intervention to support rehabilitation with CP. Finally, this review provided a record of the role of serious game-based rehabilitation for people with CP. These findings summarize the general characteristics of the overall type of interventions and conclude that the application of game-based rehabilitation in serious contexts has the potential to support long-term rehabilitation for persons with CP. This review confirms that the application of game-based rehabilitation in serious contexts may have a positive influence on the success of rehabilitation programs. Therefore, this study provides evidence to support the application of serious game-based rehabilitation for people with CP. Future research should focus on studies that use interventions concerning activity and participation, rather than physical function, for game-based rehabilitation interventions, which are necessary to provide evidence in a serious context for the application of game-based rehabilitation for persons with CP.

## Figures and Tables

**Table 1:** Number of articles by level of evidence.

Levels of Quality	Definition	Frequency (%)
I	Randomized controlled trials	5 (26.3)
II	Non-randomized two-group studies	0 (0.0)
III	Non-randomized one-group studies	6 (31.6)
IV	Single experimental studies	3 (15.8)
V	Case reports	5 (26.3)
	Total	19 (100.0)

**Table 2:** Results of McMaster Critical Review Form–quantitative studies (N = 19).

Author (Year)	Study Purpose	Literature	Design	Sample	Outcomes	Interventions	Results	Conclusions	Sum
Amengual Alcover, Jaume-i-Capó, and Moyà-Alcover [13]	1(1)	1(1)	1(1)	1(2)	2(2)	2(3)	2(4)	1(1)	11(15)
Barton, Hawken, Foster, Holmes, and Butler [14]	1(1)	1(1)	1(1)	1(2)	2(2)	2(3)	3(4)	1(1)	12(15)
Burdea et al. [41]	1(1)	1(1)	1(1)	1(2)	1(2)	2(3)	2(4)	1(1)	10(15)
Camara Machado et al. [4]	1(1)	1(1)	1(1)	1(2)	2(2)	2(3)	3(4)	1(1)	12(15)
Do, Yoo, Jung, and Park [32]	1(1)	1(1)	1(1)	1(2)	2(2)	2(3)	2(4)	1(1)	11(15)
Jaume-i-Capó, Martínez-Bueso, Moyà-Alcover, and Varona [36]	1(1)	1(1)	1(1)	1(2)	2(2)	2(3)	3(4)	1(1)	12(15)
Keller and Van Hedel [37]	1(1)	1(1)	1(1)	1(2)	2(2)	2(3)	3(4)	1(1)	12(15)
Luna-Oliva et al. [38]	1(1)	1(1)	1(1)	1(2)	2(2)	2(3)	3(4)	1(1)	12(15)
MacIntosh, Desailly, Vignais, Vigneron, and Biddiss [30]	1(1)	1(1)	1(1)	2(2)	2(2)	2(3)	3(4)	1(1)	13(15)
Moldovan, Ghircău, Podar, Rîză, and Moldovan [33]	1(1)	1(1)	1(1)	1(2)	2(2)	2(3)	2(4)	1(1)	11(15)
Peper, Van Loon, Van de Rijt, Salverda, and van Kuijk [39]	1(1)	1(1)	1(1)	1(2)	2(2)	2(3)	3(4)	1(1)	12(15)
Reifenberg et al. [34]	1(1)	1(1)	1(1)	1(2)	2(2)	2(3)	2(4)	1(1)	11(15)
Sajan, John, Grace, Sabu, and Tharion [29]	1(1)	1(1)	1(1)	2(2)	2(2)	3(3)	4(4)	1(1)	15(15)
Sandlund, Lindh Waterworth, and Häger [42]	1(1)	1(1)	1(1)	1(2)	2(2)	1(3)	3(4)	1(1)	11(15)
Sanjay, Kanitkar, Szturm, Gaonkar, and Ankolekar [35]	1(1)	1(1)	1(1)	1(2)	2(2)	2(3)	2(4)	1(1)	11(15)
Szturm et al. [40]	1(1)	1(1)	1(1)	1(2)	2(2)	2(3)	3(4)	1(1)	12(15)
Velasco et al. [31]	1(1)	1(1)	1(1)	1(2)	2(2)	3(3)	3(4)	1(1)	13(15)
Wade and Porter [15]	1(1)	1(1)	1(1)	1(2)	2(2)	3(3)	3(4)	1(1)	13(15)
Zoccolillo et al. [28]	1(1)	1(1)	1(1)	1(2)	2(2)	1(3)	4(4)	1(1)	12(15)

**Table 3:** The classification of goals according to the ICF concept.

Body Functions	Activities and Participation
Level I Balance Cervical and trunk control Motor function Functional mobility Posture control Sitting ability Upper extremity limb function Visual perceptual skills Level III Balance Gait speed Finger dexterity Motor and process skills Motor improvement Motor learning and transfer Motor performance Physical activity Postural control Running and jumping Level IV Balance Bimanual coordination Body function Upper limb motor skills Level V Impairment Long-term effects of motor function and performance Movement coupling between the trunk and pelvis Motor impairment Upper extremity motor function	Level III Activities of daily of living Level IV Activities and participations Level V Quality of life

## Data Availability

The review data used to support the findings of this study are included in the article.

## Appendix A. Evidence of Interventions in Games Used in Serious Contexts for Persons with Cerebral Palsy

**Table A1:** Evidence of serious game-based rehabilitation for persons with cerebral palsy (N = 19).

Author	Level of Evidence/Participants/Age Groups	Intervention		Outcome Measurements		Outcome
		Group	Session/Time			
Sajan, John, Grace, Sabu, and Tharion [29]	Level I N = 20 Children with CP Age groups: achool-age child, adolescent	Intervention: interactive video gaming with Nintendo Wii Control: conventional therapy	40 min, 6 days per week, 3 weeks	Static posturography as a measure of posture control Pediatric Berg’s balance scale BBT QUEST TVPS-3 Functional ambulation by walking speed and distance (endurance) measurements		Interactive video gaming with Nintendo Wii may be offered as an effective supplement to conventional therapy in the rehabilitation of children with CP
Szturm et al. [40]	Level I N = 20 Children with CP Age groups: preschooler, school-age child	Intervention: novel game-based dual-task balance exercise program Control: Conventional physical therapy balance program	45 min, 3 days per week, 12 weeks	Semi-structured interviews Pediatric Balance Scale (PBS) GMFM Computerized measures of standing balance performanc		The study demonstrates feasible trial procedures and acceptable dual-task-oriented training with a high compliance rate and positive outcomes
Velasco et al. [31]	Level I N = 10 User with CP Age groups: chool-age child	Intervention: cervical and trunk control based on serious videogames and physical exercise Control: Nnon-intervention phase	25–30 min, 10 sessions	GMFM GAS Visual analog scale Trunk control measurement scale		Physical therapy, which combines serious games with traditional rehabilitation, can help children with CP achieve better trunk and cervical functions
Wade and Porter [15]	Level I (randomized cross-over trial) N = 13 Young people with cerebral palsy Age groups: school-age child, adolescent	Intervention: a seat “cushion” containing a platform was based on a modified games controller Control: traditional physical and occupational therapy	3 months	Chailey levels of ability Sitting assessment for children with neuromotor dysfunctions		The study provides evidence that meaningful and engaging therapeutic activities using computer games controlled by upper body tilt can help to improve sitting skills in children with neuromotor dysfunctions
Zoccolillo et al. [28]	Level I (randomized cross-over trial) N = 22 Children with CP Age groups: preschooler, school-age child, adolescent	Intervention: video-game-based therapy using Xbox with Kinect device Control: conventional therapy	1 h, twice a week, 8 weeks	QUEST Abilhand kids		Video-game-based therapy using Xbox with Kinect device was effective in improving the motor functions of upper limb extremities in children with CP
Amengual Alcover, Jaume-i-Capó, and Moyà-Alcover [13]	Level III N = 9 Spastic hemiplegia and diplegia with CP Age groups: adult	Intervention: PROGame	20 min, 24 weeks	BBT FRT Tinetti balance test		These findings show significant improvements in gait and balance functions, indicators of greater independence in participating adults
Camara Machado et al. [4]	Level III N = 28 Children with CP Age groups: school-age child	Intervention: Xbox 360 Kinect games used in the rehabilitation program	40 min, twice a week, 2 months	GMFCS GMFM		The intervention led to important motor function enhancements
Jaume-i-Capó, Martínez-Bueso, Moyà-Alcover, and Varona [36]	Level III N = 9 Adults diagnosed with CP Age groups: adults	Intervention: serious games for balance rehabilitation therapy	20 min, one session per week, 24 weeks	BBS Tinetti balance test		Serious games for balance rehabilitation showed significant increases in balance and gait function scores, increasing the independence of participating adults
Keller and Van Hedel [37]	Level III N = 11 Children and adolescents with CP Age groups: school-age child, adolescent	Intervention: Armeo^®^ Spring the exergame Moorhuhn	70 min, 3 days, 2 weeks	Kinematic assessments (vertical catch 2D) BBT Five items from the Melbourne assessment		Motor learning performed to train children with CP on affected arm with weight support in a playful, virtual environment
Luna-Oliva et al. [38]	Level III N = 11 Spastic hemiplegia and diplegia with CP Age groups: school-age child	Intervention: Xbox 360 Kinect at school	30 min, 2 days a week, 2 months	AMPS Pediatric reach test 10 m walk test GMFM JHFT		Our Kinect Xbox 360 protocol showed improvements in balance and activities of daily living in CP participants in a school setting
Sandlund, Lindh Waterworth, and Häger [42]	Level III N = 14 Children with CP Age groups: school-age child	Intervention: home-based intervention using the EyeToy for PlayStation2	4 weeks	Physical activity monitors Movement assessment battery for Children-2 BOT-2 (sub-test 5, 6) 1-minute walk test Gaming diaries		Motion interactive games in a home rehabilitation setting is highly feasible to use for children with CP
Do, Yoo, Jung, and Park [32]	Level IV N = 3 Spastic hemiplegia with CP Age groups: preschooler, school-age child	Intervention: bilateral arm training using Nintendo Wii game	30 min, 12 sessions, 10 weeks	WMFT PMAL Bilateral hand coordination ability evaluation tool; putting a basketball through the hoop and moving large lightweight boxes		Bilateral coordination ability and upper limb motor skills on the affected side improved more than during the baseline period
MacIntosh, Desailly, Vignais, Vigneron, and Biddiss [30]	Level IV N = 19 Young people with CP Age groups: school-age child, adolescent	Intervention: biofeedback-enhanced therapeutic exercise video game intervention at home	60 min, once per week, 4 weeks	Active wrist extension and grip strength BBT AHA COPM Self-reported experiences of activity settings		Combining SFC-style coaching using high-quality biofeedback can complement traditional therapies by positively training adolescents in a home rehabilitation setting
Peper, Van Loon, Van de Rijt, Salverda, and van Kuijk [39]	Level IV N = 6 Spastic hemiplegia with CP Age groups: school-age child	Intervention: Lissajous-based training on bimanual performance	9 h for more than 6 weeks	AHA Rhythmic bimanual coordination Unimanual aiming		Our results evaluate the relationship between the specificity of the AHA and the expected benefits of combining the proposed training with a dedicated ambidextrous functional training program
Barton, Hawken, Foster, Holmes, and Butler [14]	Level V N = 1 Spastic diplegia with CP Age groups: school-age child	Intervention: Goblin Post Office game on the CAREN virtual rehabilitation system	30 min, twice a week, 6 weeks	GMFCS Motion of the pelvis and trunk captured in real-time by a Vicon 612 optoelectronic system tracking two clusters		Co-contractions causing increased coupling are expected to reduce over longer exposure times to training
Burdea et al. [41]	Level V N = 3 Children with CP Age groups: school-age child	Intervention: playing two custom virtual reality games	3 times per week, 12 weeks	Impairment (DF/PF torques, DF initial contact angle, and gait speed) GMFM Peds QL		Game technology is suitable for the age group and is accepted by the participants, supporting the hypothesis that the game-based robotic training of the ankle is beneficial for walking in children with CP
Moldovan, Ghircău, Podar, Rîză, and Moldovan [33]	Level V N = 1 Spastic hemiplegia with CP Age groups: school-age child	Intervention: MIRA exergames with Kinect Xbox^®^ sensor	30 min, 3 sessions a week, 14 months	ABILHAND kids QUEST Pediatric balance scale MIRA testing schedule		Long-term virtual occupational therapy associated with the conventional rehabilitation program improved motor function and performance in a child with cerebral palsy and hemiparesis
Reifenberg et al. [34]	Level V N = 1 Spastic hemiplegia with CP Age groups: preschooler	Intervention: game-based neurorehabilitation using telehealth technologies	7 h for 8 weeks	AHA BOT-2 QUEST PMAL PEDI-computer adapted test Perceived stress scale	It is feasible to administer game-based neurorehabilitation to a child with cerebral palsy	
Sanjay, Kanitkar, Szturm, Gaonkar, and Ankolekar [35]	Level V N = 1 Left spastic hemiplegia with CP Age groups: preschooler	Intervention: computer game-assisted repetitive task practice-based upper extremity therapy program	40–60 min, 2~3 days per week, 16 weeks	PDMS-2 QUEST Computerized assessment of a broad range of object manipulation tasks	The feasibility and acceptability of the G-RTP program demonstrated for use by children with UE motor impairments	

AHA: Assisting Hand Assessment, AMPS: Assessment of Motor and Process Skills, BBS: Berg Balance Scale, BOT-2: Bruininks-Oseretsky Test of Motor Proficiency—Second Edition, COPM: Canadian Occupational Performance Measure, FRT: Functional Reach Test, GAS: Goal Attainment Scale, GMFCS: Gross Motor Function Classification System, GMFM: Gross Motor Function Measure, JHFT: Jebsen Taylor Test of Hand Function, PEDI: Pediatric Evaluation and Disability Inventory, PDMS-2: Peabody Developmental Motor Scales—Second Edition, PMAL: Pediatric Motor Activity Log, QUEST: Quality of Upper Extremity Skills Test, TVPS-3: Test for Visual-Perceptual Skills—Third Edition, WMFT: Wolf Motor Function Test.
